# Assessment of the Level of Physical Activity and Body Mass Index of Soldiers of the Polish Air Force

**DOI:** 10.3390/ijerph19148392

**Published:** 2022-07-09

**Authors:** Andrzej Tomczak, Anna Anyżewska, Jerzy Bertrandt, Tomasz Lepionka, Artur Kruszewski, Agata Gaździńska

**Affiliations:** 1 Military Section, Polish Scientific Physical Education Association, 00-809 Warsaw, Poland; 2 University of Economics and Human Sciences in Warsaw, 59 Okopowa, 01-043 Warsaw, Poland; a.anyzewska@vizja.pl; 3 Faculty of Economic Sciences, John Paul II University of Applied Sciences in Biała Podlaska, 95/97 Sidorska, 21-500 Biała Podlaska, Poland; jwbertrandt@gmail.com; 4 Biological Threats Identification and Countermeasure Center, Military Institute of Hygiene and Epidemiology, 4 Kozielska, 01-163 Warsaw, Poland; tomasz.lepionka@wihe.pl; 5 Department of Individual Sports, Józef Piłsudski University of Physical Education in Warsaw, Marymoncka 34 St., 01-813 Warsaw, Poland; artur.kruszewski@awf.edu.pl; 6 Laboratory of Dietetics and Obesity Treatment, Department of Psychophysiological Measurements and Human Factor, Research Military Institute of Aviation Medicine, 54/56 Krasińskiego, 01-755 Warsaw, Poland; afrotena@gmail.com

**Keywords:** IPAQ, soldiers, military pilots, physical activity, BMI

## Abstract

Level of physical activity positively affects health condition, correlates highly with level of physical fitness and contributes to the efficient performance of military tasks. The aim of the study was to assess the level of physical activity and body mass index of the Polish Air Force soldiers. A total of 543 professional soldiers (men) doing military service in military units of the Polish Air Force underwent the examination. The average age of examined soldiers amounted to 34.8 ± 9.0 years. In order to carry out the research, the long version of the International Physical Activity Questionnaire was used. Out of the total of 477 questionnaires that qualified for the analysis, a high level of physical activity was found among 81.1% of subjects, moderate 10.5% and low 8.4%. Average MET values were obtained in the following: job-related, relocation (transportation), housekeeping, recreation (leisure activities and sport). The average MET values were 4173 ± 5306 MET; 2371 ± 2725 MET; 2455 ± 4843 MET; 2421 ± 2802 MET, respectively. The average level of body mass index amounted to 25.98 ± 3.38 kg/m^2^. The tested Air Force soldiers were generally characterized by a high and moderate level of physical activity. Generally, there was no difference in the level of physical activity between the flight crew and the ground staff.

## 1. Introduction

It is generally believed that level of physical activity (PA) largely reflects state of health, as reflected in the saying “healthy body, healthy mind”. Numerous studies have proven that, among other things, lack of physical activity contributes to the occurrence and development of many diseases [1,2,3,4]. Therefore, much attention is paid to promoting physical activity and pro-health lifestyles in society for everyone, from children to older people. Deficiencies in physical activity caused by civilization should be reduced by conscious participation in physical activities.

In the uniformed services, a high level of physical fitness is very desirable in the aspects of professional preparation and efficient fulfilling of service tasks (e.g., a soldier on the battlefield, a policeman during an intervention or a firefighter during a rescue operation) [5,6]. The way to achieve the desired level of physical fitness for a soldier (an officer of a particular formation) is participation in various forms of physical activity during work and leisure time. This should be reflected in a high level of physical activity. Soldiers, as part of their professional work, should participate in obligatory physical education classes, diversified by the number of hours depending on military specialty [7].

The group of soldiers in the Air Force includes soldiers of various specialties, most of which specialties are also in both the Navy and the Land Forces. These specialties are, for example, administration or logistics. Even such specialties as ground service of aviation equipment (engineering and aviation service) occurs in other branches of armed forces. In the Air Force there are particular specialties typical of military air services, like that of a supersonic airplane pilot. There is the main division of the flight crew and ground staff. The main differences are the environment of performing official tasks (air, land) and the way of logging in to perform them [8]. These differences also apply to physical preparation, in terms of the volume of classes and means of physical education (i.e., physical exercises).

In the literature, we can find many papers on the physical fitness of soldiers [9,10,11]. So far, not much work has been published regarding the assessment of the physical activity level of Polish soldiers, and those from other countries. In Poland, comparative research has been conducted among soldiers of military administration, special units, soldiers of the Land Forces Training Center, soldiers of the Land Forces and among soldiers of the special unit of the Military Police, and there have been assessments of physical activity level carried out among candidates seeking to become professional soldiers [12,13,14,15,16]. In general, a high level of physical activity was revealed for soldiers. However, a small percentage of soldiers also revealed a low level of it, which is not a good predictor of efficient performance of service duties. Mierzejewski conducted research on the level of physical activity among soldiers of land troops qualified for the Officer’s Study. He stated that 79.0% of the respondents presented a high level of physical activity and 21.0% a moderate level [14]. In turn, Tomczak et al. conducted research using the International Physical Activity Questionnaire (IPAQ) tool among Polish soldiers of the Military Police. Based on the research, they found that 84.6% of the respondents were characterized by a high level of physical activity, 7.7% were moderate, and 7.7% were low [15]. Comparative research on the physical activity among soldiers of the National Reserve Force was carried out by Szettler-Degler [17]. She concluded that the level of metabolic equivalent (MET) was similar between men and women. Łyżwiński, on the basis of his own study, found that 80.0% of the surveyed Polish soldiers of the land forces perceived there to be a relationship between physical fitness and better performance of official tasks. More than half of the respondents revealed that they undertook physical activity outside of business hours and that they assessed their physical fitness as very good [13]. In many works, attention is paid to the need to raise awareness of the necessity to undertake physical activity and to motivate people to undertake physical activity [15,18,19].

The main aim of the research presented here was to assess the level of physical activity and the body mass index (BMI) of soldiers of the Polish Air Force. The specific objectives were to identify differences in the levels of physical activity and BMI between flight crew and ground staff and age groups (up to 30 years of age, 31–40 years of age and a group over 40). We hypothesized that the flight crew would reveal a higher level of physical activity than ground staff and that a higher level of physical activity would be presented by younger soldiers. Our hypothesis regarding flight crew versus ground staff was justified by the fact that flight crew participate in more compulsory physical education and recreation classes than other soldiers.

## 2. Materials and Methods

### 2.1. Study Design and Participations

A total of 543 professional soldiers (men) doing military service in military units of the Polish Air Force underwent the examination. The subjects were selected randomly. The soldiers performed professional military service in 35 military units of the Air Force. The research sample accounted for about 3% of the population of soldiers of the Air Force. The average age of examined soldiers amounted to 34.8 ± 9.0 (19–59) years old. Due to the failure to meet the criteria of credibility, 66 questionnaires did not qualify for further studies. The percentage of questionnaires that qualified for the analysis was 87.8%.

The results of the research were presented in two basic groups, i.e., flight crew 37.49 ± 7.54 (21–59) years old and ground staff 32.70 ± 9.57 (19–59) years old. The flight crew included military pilots, on-board technicians, and on-board navigators, whereas the ground staff included ground service of the aircraft, engineers, technicians, and staff soldiers. The justification for such division was the environment of performing main official tasks (flight crew—in the air; ground staff—on the ground). Subsequently, the research material was divided into the following age groups: up to 30 years old, 31–40 years old and a group over 40 years old. The age of the respondents was determined based on date of birth.

The research was carried out in 2017–2018 as part of the National Health Program research project. The permission of the Bioethics Committee of the Military Institute of Hygiene and Epidemiology in Warsaw (Poland) was obtained for conducting the research (No 01/2016). All procedures were performed in accordance with the ethical standards as laid down in the 1964 Declaration of Helsinki and its later amendments or comparable ethical standards. All participants provided informed consent.

### 2.2. Physical Activity Assessment

In order to carry out the research, the long version of IPAQ was used. In the introduction to the questionnaire a vigorous physical activity and moderate physical activity were defined in a way accessible for everybody. Vigorous physical activity is understood as heavy physical exercise that forces strong, increased breathing, and, therefore, accelerated heart rate. Moderate physical activity is understood as an average effort with slightly increased breathing and a little accelerated heart action [20,21]. The first part of the questionnaire dealt with physical efforts related to professional work (Part 1: Job-related PA). Three types of efforts were distinguished, i.e., vigorous, moderate and walking. The second part dealt with physical efforts related to relocating (walking, cycling; Part 2: Transportation (relocation) physical activity). In the third part, subjects were asked about the time they spent on taking physical activity related to housework, general housekeeping and for family care (Part 3: Housework, house maintenance, and caring for family). The fourth part of the questionnaire was connected with recreation, sport and physical activity in leisure time (Part 4: Recreation, sport, and leisure-time physical activity). The fifth part contained questions related to time spent sitting, divided into weekend and week days (Part 5: Time spent sitting).

Based on the number of days, time spent on given physical activity and MET rate determined for a given physical activity, a total energy expenditure for each type of physical exercise was calculated. 1 MET corresponds to the average energy expenditure during rest (in a sitting position). It is equivalent to the consumption of oxygen in a sitting position of a person weighing 70 kg, which is 3.5 mL O₂/min/kg [22].

### 2.3. Somatic Measurements

The measurement of basic morphological features was based on the analysis of the following values of somatic measurements, which were measured according to the Martin technique, body height, and body mass [23]. Body height was measured with an anthropometer (Holtain, UK) to the nearest 1 mm, in a standing upright position, without shoes. Body mass was measured using a Charder MS 4202L electronic weight floor scale (measurement accuracy < 50 g). Each measurement was performed under the same test conditions (in the morning, about 30 min before the first meal). The respondent was wearing sports clothes and barefoot. The same measuring equipment was used during all stages of the research, the accuracy of which was checked periodically. Measurements were made by people who graduated in physical education.

Based on these data, the BMI was calculated (BMI = body mass/height^2^ [kg/m^2^]). The scale of BMI classification reported by the World Health Organization (WHO) was accepted [24].

### 2.4. Statistical Analysis

Descriptive statistics were means (±standard deviations), medians, quartiles 1 and 3, as well as interquartile range. Normality checks were done with the Shapiro–Wilk test. To compare groups, we used the Mann–Whitney test, as well as the Kruskall-Wallis test. The Chi-square test, to relate nominal data, and the Spearman correlation, and its 95% CI, were also used. Effect sizes were calculated (partial eta square, and Cramer’s V). These analyses were done in Statistica v.13.3 (Statsoft, Kraków, Poland), and the significance level was set at 5%. Further sample size estimation was done with the G*Power v. 3.1. software (Heinrich-Heine-Universität, Düsseldorf, Germany).

## 3. Results

The calculations using the G*Power test were made a posteriori. The minimum sample size should be 220 subjects for the Chi-square test (effect size = 0.3; power = 0.95). The minimum sample size should be 252 subjects for the Kruskal–Wallis test (effect size = 0.25; power = 0.95). In our research we met these requirements with a significant surplus, as 477 questionnaires were qualified for the analysis.

Out of the total of 477 questionnaires which qualified for the analysis, a high level of physical activity was found among 81.8% (*n* = 390) of subjects, a moderate level in 10.2% (*n* = 50) and low in 8.0% (*n* = 40). Taking into account only the flight crew, it was found that a high level of physical activity characterized 80.9% of the soldiers, moderate 11.9%, and low 7.2%. In the ground staff, the following results were obtained: 82.4%, 9.0% and 8.6%, respectively (Table 1).

Table 1 presents the results of the Chi-square test for the levels of physical activity depending on the type of specialization and the age of the respondents. The analysis showed no association between specialization and the level of physical activity. The relationship between age and the level of physical activity among flight crew also turned out to be insignificant. A significant association of low strength was noted between the level of physical activity and the age of ground staff. In the age group under 30, the percentage of soldiers with a high level of physical activity was significantly higher than in the other two age groups (90.0% vs. 78.3% and 75.4%). The percentage of soldiers up to 30 years of age with a low level of physical activity was significantly lower than among soldiers aged 31–40 (2.7% vs. 14.1%).

BMI was calculated for professional role and in terms of age groups of the soldiers (Table 2). The average BMI for all Air Force soldiers was 25.98 kg/m^2^, for flight crew 26.65 kg/m^2^, and for ground staff 25.46 kg/m^2^. Normal body weight was revealed by 40.5% of all soldiers of the Air Force (flight crew 34.3%, ground staff 45.4%). Overweight was found in 46.5% of soldiers (flight crew 46.7%, ground staff 46.4%). On the other hand, obesity was revealed by 12.8% of all soldiers of the Air Force (flight crew19.0%, ground staff 7.9%), while underweight was diagnosed in one soldier (ground staff). Among the flight crew, the highest percentage of soldiers with normal body weight was found in soldiers under 30 (52.2%). The highest percentage of overweight soldiers was found among soldiers in the 31–40 age group (56.7%). On the other hand, soldiers in the age group over 40 showed the highest percentage of obesity (28.6%). The same tendency was found in the group of ground staff: normal body weight had the highest percentage among the youngest soldiers (68.2%), the highest percentage of overweight soldiers was in the 31–40 age group (58.7%), and obese soldiers were in the age group above 40 (16.9%).

In each age group of flight crew, a higher percentage of obese soldiers was found (statistically significant difference). Statistically significant differences in BMI values were found between the flight crew and ground staff. The following regularity was observed, BMI increases with the age of the subjects. The highest BMI was found in the group of flight crew who were over 40 (BMI = 27.67 kg/m^2^).

In the next stage of the analysis of the research results, the total MET value, and the MET value for individual types of physical activity, were calculated (in accordance with the parts listed in IPAQ). It was found that the overall MET level in the group of ground staff was higher than that of the flight crew (12,333 MET vs. 10,262 MET; *p* < 0.05). For the activity from Part 1: Job-related PA, a statistically significant difference in MET values between the flight crew and ground staff was found (respectively: 3367 MET vs. 4807 MET). In the remaining parts of the listed activities, i.e., transportation of PA, housework, leisure-time PA, no statistically significant differences were found (Table 3).

Table A1 presents the results of the studies on the total MET value for each age group. In the group of flight crew, the highest total MET value (10,625 MET) was disclosed by soldiers over 40. However, in the group of ground staff, the highest MET value was for soldiers aged 31–40 (13,045 MET). The MET values calculated for the physical activities listed in the parts of the questionnaire (job-related PA, transportation PA, housework, leisure time) in individual age groups were also subjected to detailed analysis (Table A2, Table A3, Table A4 and Table A5). Taking into account the total number of Air Forces soldiers, the highest MET values related to professional work, transportation (relocation) and housework were revealed by soldiers of the age group up to 30 (respectively: 5059 MET; 2653 MET; 2959 MET) (Table A3, Table A4 and Table A5). Flight crew over 40 received the highest MET value related to leisure time (2559 MET) (Table A5). A comparison was also made between the groups of flight crew and ground staff, MET values for intensive efforts, MET values for moderate efforts and MET values for walking. The flight crew obtained 3100 MET for intensive efforts, while the ground staff attained 3900 MET. For moderate efforts, 3214 MET was obtained by the flight crew and 3534 MET by the ground staff. For walking the results were, respectively: 4008 MET and 4871 MET (all statistically significant differences).

There were no statistically significant differences between the surveyed groups of soldiers in the variables “time spent sitting on a weekday” and “time spent sitting on a weekend”. It was calculated that the flight crew spends 258 min per weekday and 211 min per weekend sitting (ground staff: 235 min and 219 min, respectively).

In order to determine which variables had the greatest impact on BMI, correlations were calculated and the level of statistical significance was determined. No correlations were found in the group of All Air Force soldiers (Table 4). Taking into account the total number of All flight crew, a statistically very weak negative correlation was found between BMI and total MET, MET work, MET transportation (relocation) and walking. In the group of ground staff only one correlation was found, between BMI and MET work (<30 years).

## 4. Discussion

It was hypothesized that a higher level of physical activity would be revealed by soldiers belonging to the group of flight crew. The premise for such a hypothesis was the fact that more attention is paid to physical preparation in the flight crew. This was manifested in an increased number of physical education classes (flight crew at least 6 h per week, ground staff at least 4 h per week), and the fact that the flight crew participated once a year in three-week training and fitness camps [25]. On working days during training and fitness camps, a minimum of 6 h of physical education, sports and recreation classes were carried out. Wider educational and promotional activities in the field of a healthy lifestyle were also undertaken in relation to the flight crew. The Military Institute of Aviation Medicine plays a leading role in this aspect. Based on the results of the studies, the hypothesis was rejected. It turned out that it was the ground staff who represented a higher level of physical activity (expressed as total MET). Another surprising result was the disclosure of the fact that 8% of the Air Force soldiers were classified in a group with a low level of physical activity. This is a worrying percentage as this occupational group is expected to have a high, or at least moderate, level of physical activity. It turned out that a similar percentage of soldiers with low levels of physical activity occurred in the group of flight crew and ground staff (7.2% and 8.6%, respectively). A similar result was obtained by Tomczak et al. (2011) in studies conducted among military police soldiers (7.7%) [15]. In turn, Mierzejewski, in the research carried out at the Land Forces Training Center, did not find any soldier with a low level of physical activity [14]. This may suggest that as long as soldiers participate in military training in barracks, they are ensured an adequate level of physical activity by participation in varied military training. However, after returning to military units and everyday life, the issues of physical activity become less important.

Analyzing the levels of physical activity in individual age groups, a disturbing phenomenon was observed. In accordance with the general trend, it was assumed that younger soldiers and flight crew would be characterized by a higher level of physical activity. This assumption was also not confirmed. Among flight crew aged up to 30, a lower percentage of soldiers with a high level of physical activity was revealed than in the age group up to 30 of ground staff (81.8% and 90.0%, respectively). The highest percentage of soldiers with low levels of physical activity was also revealed among flight crew up to the age of 30 (9.1%) and ground staff (2.7%).

The level of physical activity is related to the value of BMI, greater physical activity is one of the factors influencing the reduction of body mass [26]. Assuming the BMI criteria, all the Air Force soldiers were revealed to be slightly overweight (25.98 kg/m^2^). BMI increased with the age of the respondents. Higher BMI was found in the flight crew (Table 2). However, BMI should be interpreted with great caution in relation to athletes (strength competitions), as well as physically active people who regularly practice strength exercises, because then we may have to deal with the so-called muscle overweight [27]. Interesting information is provided by a comparative analysis of the currently obtained BMI results of flight crew with the results of research conducted fifteen years ago by Henrykowska and Tomczak, also among the polish flight crew [28]. At that time, the level of BMI of flight crew was 25.43 kg/m^2^, which was 1.22 units lower than at present. For soldiers in the group up to 30 years old, the BMI level was 23.50 kg/m^2^ (it was lower by 1.55 units), for soldiers in the group 31–40 years old, 24.41 kg/m^2^ (it was lower by 1.65 units), and for the group of soldiers over 40, 27.99 kg/m^2^ (it was lower by 0.32 units) [28]. Definitely higher values currently occur in the group of respondents up to the age of 30 and in the group aged 31–40. These research results show an unfavorable trend taking place, not only in the military environment [29].

Many researchers have studied the influence of BMI level on injuries suffered by soldiers [30]. There is insufficient scientific evidence for BMI in general as a modifiable risk factor. However, there is strong scientific evidence for obesity, for being overweight and underweight, as a modifiable risk factor for musculoskeletal injuries [31,32,33]. It was also found that soldiers who were older had higher BMIs, ran longer distances during unit physical training, and had lower cardiovascular endurance as measured by the two-mile run and were at a higher risk of a running-related injury [33].

A result worth analyzing is the MET value during professional work (IPAQ Part I). A significantly higher MET value was found for the ground staff compared to the flight crew (4807 MET and 3367 MET, respectively). The flight crew is generally believed to perform heavier work when performing tasks in the air. However, this was not confirmed by the conducted surveys. Such a result was most likely influenced by an increase in the degree of technicality of military equipment and the associated change in the manner of performing work (loads, performance of activities). As noted in the modern world, coordination skills are more important than fitness skills [34,35]. In spite of these changes, the ground staff, which consisted of technicians operating aircraft, performs many activities requiring effort (moving equipment, lifting, moving as walking or cycling).

The present considerations lead to a reflection that educational errors, related to the promotion of a healthy lifestyle, may have occurred in military units. Kaiser and Sokołowski stated that the army as a total institution has a significant impact on the lifestyle (pro-health attitudes) of soldiers. Therefore, it would be advisable to modify the existing military school programs by taking into account the issues of health education, so that the superiors not only acquire command skills, but also become model health educators [12]. Mullie et al. (2013), on the other hand, indicated that the army, instead of relying on civilian actions in the field of public health, should develop its own specific methods of preventing weight gain, improving physical fitness and influencing attitude to smoking [36].

### Limitations

As stated by the Polish researchers, the respondents’ self-completion of the questionnaires led to some overestimation of the type and duration of efforts [37]. So, this seems to be the biggest limitation of our research. However, it should be emphasized that the respondents were instructed in detail before filling in the questionnaires. When filling in the questionnaires, the respondents had the opportunity to ask the interviewer questions. Another limitation reported by researchers is the use of the long version of IPAQ instead of the short version. The respondent is forced to spend a longer time completing the questionnaire. In our opinion, it does not seem that the time needed to complete the IPAQ was long enough to discourage respondents from filling in the questionnaire diligently. Moreover, the long version of the IPAQ is recommended in scientific research, especially when one of the forms of physical activity is carefully analyzed [38]. In our research, we tried to carefully analyze various forms of physical activity in different professional roles and age groups.

## 5. Conclusions

In general, the Air Force soldiers represented a high and moderate level of physical activity. However, every fifteenth respondent revealed a low level of physical activity. There were no differences in the level of physical activity between flight crew and ground staff. Moreover, the BMI of the examined soldiers indicated overweight tendencies, and a comparative analysis with the results of research from many years ago revealed that the largest increase in BMI occurred in the group of soldiers aged 19 to 30. In the Air Force, educational programs on health promotion specific to the Air Force and individual military specialties should be introduced. Preventive programs to date have not contributed to an increase in care regarding the correct level of body weight, and to an increase in level of awareness regarding the positive aspects of physical activity. The above statements were particularly strongly confirmed by the results of research on soldiers of the youngest age (up to the age of 30). It is therefore all the more necessary to undertake immediate educational interventions.

## Figures and Tables

**Table 1:** Summary of the levels of physical activity of the Air Force soldiers according to the IPAQ classification.

	Level of Physical Activity					
Variables	High	Moderate	Low	χ^2^	*p*-Value	V
All Air Force soldiers (*n* = 477)	81.8% (390)	10.2% (49)	8.0% (38)			
All flight crew (*n* = 210)	80.9% (170)	11.9% (25)	7.2% (15)	1.63	0.442	0.06
All ground staff (*n* = 267)	82.4% (220)	9.0% (24)	8.6% (23)			
Flight crew for years 30 (*n* = 44)	81.8% (36)	9.1% (4)	9.1% (4)	1.86	0.761	0.07
Flight crew 31–40 years old (*n* = 96)	83.3% (80)	10.4% (10)	6.3% (6)			
Flight crew above 40’s (*n* = 70)	77.1% (54)	15.8% (11)	7.1% (5)			
Ground staff for years 30 (*n* = 110)	90.0% (99) _b_	7.3% (8) _a_	2.7% (3) _a_	12.38	0.015	0.15
Ground staff 31–40 years old (*n* = 92)	78.3% (72) _a_	7.6% (7) _a_	14.1% (13) _b_			
Ground staff above 40’s (*n* = 65)	75.4% (49) _a_	13.8% (9) _a_	10.8% (7) _a,b_			

The values in a given column not sharing a letter index between rows differ at the *p* < 0.05 (Bonferroni correction). Legend: V—effect size.

**Table 2:** Summary of the BMI of the subjects [kg/m^2^].

BMI for:	Age								
	<30 Years		31–40 Years		>40 Years		H	*p*-Value	η^2^
	Me	IQR	Me	IQR	Me	IQR			
All Air Force soldier	24.19 _a_	3.31	26.26 _b_	3.73	26.83 _b_	4.94	76.74	<0.001	0.16
Flight crew	24.71 _a_	4.35	26.25 _b_	3.64	27.07 _b_	5.58	13.77	<0.001	0.06
Ground staff	23.96 _a_	3.11	26.24 _b_	3.72	26.57 _b_	4.10	58.07	<0.001	0.21

The values in a given column not sharing a letter index between rows differ at the *p* < 0.05 (Bonferroni correction). Legend: IQR—interquartile range; Me—median; H—Kruskal-Wallis test statistic; η^2^—effect size.

**Table 3:** Summary of MET values in flight crew and ground staff according to IPAQ criteria.

Variable	All Flight Crew (*n* = 210)		All Ground Staff (*n* = 267)				
	X ± SD	Median	X ± SD	Median	Z	*p*-Value	r_g_
Total MET	10,262 ± 10,330 ^#^	7456	12,333 ± 9841	10,770	−3.05	*p* = 0.002	0.14
MET total work (part 1)	3367 ± 4334 ^#^	1893	4807 ± 5892	3270	−2.69	*p* = 0.007	0.12
MET total relocation (part 2)	2160 ± 2300	2160	2539 ± 3011	1512	−0.91	*p* = 0.361	0.04
MET total homework (part 3)	2648 ± 6116	1167	2303 ± 3539	2303	0.37	*p* = 0.712	0.02
MET total leisure time (part 4)	2087 ± 2119	1596	2684 ± 3219	1600	−1.17	*p* = 0.243	0.05

^#^ statistically significant differences on the level *p* < 0.05. part 1, 2, 3, 4—parts of IPAQ; r_g_—effect size.

**Table 4:** Correlation of BMI with other variables related to IPAQ.

BMI [kg/m^2^]:		All Flight Crew (*n* = 210)			All Ground Staff (*n* = 267)	
	r	CI 95%	*p*-Value	r	CI 95%	*p*-Value
Total MET	−0.23	−0.35; −0.09	<0.001			
	−0.42 ^#^	−0.64; −0.13	0.005	-		
MET total work (IPAQ part 1)	−0.16	−0.29; −0.02	0.020	0.27 ^#^	0.08; 0.44	0.004
	−0.31 ^#^	−0.56; −0.002	0.050			
MET total transportation (IPAQ part 2)	−0.14	−0.17; 0.004	0.027			
MET walking	−0.19	−0.32; −0.05	0.006			

Legend: ^#^ only soldiers in the age group for 30 years old; part 1, 2—parts of IPAQ; CI—confidence intervals.

## Data Availability

Data sharing is not applicable to this article.

## Appendix A

**Table A1:** Comparison of the total MET (professional work, transportation, housework, leisure time).

No.	Variables	Mean ± SD	Median	Q1; Q3 (Quartile Deviation)	Coef. Var.
1	Air Force soldiers for years 30 (*n* = 154)	13,175 ± 11,660	10,224	5282; 17,538 (6128)	88
2	Air Force soldiers 31–40 years old (*n* = 188)	10,367 ± 8642	8714	3727; 14,214 (5243)	83
3	Air Force soldiers above 40’s (*n* = 135)	10,889 ± 8642	8586	4905; 14,096 (4596)	91
4	Flight crew for years 30 (*n* = 44)	10,541 ± 11,376	7641	4941; 12,354 (3706)	108
5	Flight crew 31–40 years old (*n* = 96)	9870 ± 8514	7378	3727; 13,077 (4675)	86
6	Flight crew above 40’s (*n* = 70)	10,625 ± 11,933	7432	4287; 12,195 (3954)	112
7	Ground staff for years 30 (*n* = 110)	12,871 ± 9624	10,864	6447; 16,000 (4776)	75
8	Ground staff 31–40 years old (*n* = 92)	13,045 ± 10,709	11,740	4237; 21,403 (8583)	82
9	Ground staff above 40’s (*n* = 65)	10,415 ± 8760	8202	3012; 16,730 (6859)	84

**Table A2:** Summary of total MET related to professional work.

No.	Variables	Mean ± SD	Median	Q1; Q3 (Quartile Deviation)	Coef. Var.
1	Air Force soldiers for years 30 (*n* = 154) ^	5059 ± 6786	2833	1.0; 7371 (3685)	134
2	Air Force soldiers 31–40 years old (*n* = 188)	3523 ± 4436	1879	99; 4830 (2365)	126
3	Air Force soldiers above 40’s (*n* = 135)	4068 ± 4301	3150	693; 5760 (2533)	106
4	Flight crew for years 30 (*n* = 44)	3424 ± 3920	2607	346; 4665 (2159)	114
5	Flight crew 31–40 years old (*n* = 96)	3412 ± 4749	1488	313; 4581 (2134)	139
6	Flight crew above 40’s (*n* = 70)	3268 ± 4030	1980	537; 4476 (1965)	123
7	Ground staff for years 30 (*n* = 110)	5413 ± 6800	4116	1485; 7518 (3016)	126
8	Ground staff 31–40 years old (*n* = 92)	4569 ± 5223	2617	1.0; 7612 (3806)	114
9	Ground staff above 40’s (*n* = 65)	4120 ± 5067	2160	0.0 ± 5850 (2925)	123

^ The difference at a statistically significant level between the groups No 1–2 (*p* < 0.05).

**Table A3:** Summary of total MET related to relocate in different groups.

No.	Variables	Mean ± SD	Median	Q1; Q3 (Quartile Deviation)	Coef. Var.
1	Air Force soldiers for years 30 (*n* = 154) ^	2653 ± 2932	1758	495; 3600 (1552)	111
2	Air Force soldiers 31–40 years old (*n* = 188)	2245 ± 2830	1350	396; 3492 (1548)	126
3	Air Force soldiers above 40’s (*n* = 135)	2228 ± 2290	1638	495; 2988 (1246)	103
4	Flight crew for years 30 (*n* = 44)	2181 ± 2472	1386	396; 3130 (1367)	113
5	Flight crew 31–40 years old (*n* = 96)	2058 ± 2070	1386	448; 3501 (1526)	101
6	Flight crew above 40’s (*n* = 70)	2287 ± 2508	1674	318; 2952 (1254)	110
7	Ground staff for years 30 (*n* = 110)	2730 ± 3397	1592	693; 3483 (1741)	124
8	Ground staff 31–40 years old (*n* = 92)	2696 ± 3040	2065	144; 3742 (1799)	113
9	Ground staff above 40’s (*n* = 65)	1994 ± 2116	1116	396; 2799 (1201)	160

^ The difference at a statistically significant level between the groups No. 1–3 (*p* < 0.05).

**Table A4:** Summary of total MET related to housework.

No.	Variables	Mean ± SD	Median	Q1; Q3	Coef. Var.
1	Air Force soldiers for years 30 (*n* = 154)	2959 ± 6403	1260	330; 4020	216
2	Air Force soldiers 31–40 years old (*n* = 188)	2346 ± 2801	1440	390; 3400	119
3	Air Force soldiers above 40’s (*n* = 135)	2033 ± 5006	810	0.0; 2460	246
4	Flight crew for years 30 (*n* = 44)	3088 ± 9574	990	422; 2415	310
5	Flight crew 31–40 years old (*n* = 96)	2356 ± 2815	1305	435; 3240	120
6	Flight crew above 40’s (*n* = 70)	2773 ± 6708	1080	180; 3060	242
7	Ground staff for years 30 (*n* = 110) ^	1742 ± 1956	940	90; 3240	112
8	Ground staff 31–40 years old (*n* = 92)	2981 ± 5200	1590	0.0; 3690	174
9	Ground staff above 40’s (*n* = 65)	2294 ± 2436	1380	360; 4200	106

^ The difference at a statistically significant level between the groups No. 7–8 (*p* < 0.05).

**Table A5:** Summary of total MET related to leisure time.

No.	Variables	Mean ± SD	Median	Q1; Q3 (Quartile Deviation)	Coef. Var.
1	Air Force soldiers for years 30 (*n* = 154)	2504 ± 2929	1440	438; 3531 (1546)	117
2	Air Force soldiers 31–40 years old (*n* = 188)	2253 ± 2890	1555	305; 3063 (1379)	128
3	Air Force soldiers above 40’s (*n* = 135)	2559 ± 2524	1986	546; 3828 (1641)	99
4	Flight crew for years 30 (*n* = 44)	1848 ± 2081	1278	313; 2754 (1220)	113
5	Flight crew 31–40 years old (*n* = 96)	2044 ± 2017	1645	519; 2790 (1135)	99
6	Flight crew above 40’s (*n* = 70)	2296 ± 2284	1765	206; 3744 (1769)	99
7	Ground staff for years 30 (*n* = 110) ^	2986 ± 3468	1815	914; 4273 (1680)	116
8	Ground staff 31–40 years old (*n* = 92)	2798 ± 3225	1551	148; 4470 (2161)	115
9	Ground staff above 40’s (*n* = 65)	2007 ± 2674	1116	1.0; 3051 (1525)	133

^ The difference at a statistically significant level between the groups No. 7–9 (*p* < 0.05).
